# Dynamic changes and clinical significance of serum S100B protein and glial fibrillary acidic protein in patients with delayed encephalopathy after acute carbon monoxide poisoning

**DOI:** 10.12669/pjms.344.15363

**Published:** 2018

**Authors:** Chong Di, Yun Zeng, Jingyu Mao, Zhengjie Shen, Wenzhe Gu

**Affiliations:** 1 Chong Di, Emergency Center, Affiliated Hospital of Xuzhou Medical University, Xuzhou 221006, Jiangsu Province, China; 2 Yun Zeng, Department of Medical Oncology, Jiangsu Cancer Hospital, Nanjing 210009, Jiangsu Province, China Jiangsu Collaborative Innovation Center of Traditional Chinese Medicine (TCM) Prevention and Treatment of Tumor, Nanjing University of Chinese Medicine, Nanjing 210023, Jiangsu Province, China; 3 Jingyu Mao, Jiangsu Collaborative Innovation Center of Traditional Chinese Medicine (TCM) Prevention and Treatment of Tumor, Nanjing University of Chinese Medicine, Nanjing 210023, Jiangsu Province, China; 4 Zhengjie Shen, Medical Oncology of Zhangjiagang First People’s Hospital, Zhangjiagang 215600, Jiangsu Province, China; 5 Wenzhe Gu, Depr. of Otorhinolaryngology, Zhangjiagang Hospital of Traditional Chinese Medicine, Zhangjiagang 215600, Jiangsu Province, China

**Keywords:** S100B, Glial fibrillary acidic protein, Delayed encephalopathy, Acute carbon monoxide poisoning

## Abstract

**Objective:**

To study the dynamic changes and clinical significance of serum S100B protein and glial fibrillary acidic protein (GFAP) in patients with delayed encephalopathy after acute carbon monoxide poisoning (DEACMP).

**Methods:**

This study was conducted among DEACMP patients who were hospitalized from November 2014 to February 2016. Serum levels of S100B and GFAP in 66 DEACMP patients were measured by ELISA. Changes in patient states were examined dynamically using activities of daily living (ADL) scale, information-memory-concentration test (IMCT) and Hasegawa’s dementia scale (HDS), and compared with those of 64 patients without DE after ACMP.

**Results:**

Serum S100B [(0.59 ± 0.11) ng/ml] and GFAP [(227.67 ± 12.43) ng/ml] levels of DEACMP group in acute phase were significantly higher than those of ACMP group [(0.48 ± 0.10) ng/ml and (178.91 ± 11.47) ng/ml] and DEACMP group in recovery phase [(0.49 ± 0.12) ng/ml and (179.54 ± 12.32) ng/ml] (all P<0.05). Serum S100B and GFAP levels of DEACMP group were significantly correlated in both acute and recovery phases (r=0.432 in acute phase, P=0.007; r=0.378 in recovery phase, P=0.034). ADL, HDS and IMCT scores of DEACMP group in acute phase were (45.12 ± 3.12), (7.98 ± 1.02) and (9.61 ± 1.41) points respectively, which were significantly different from those of recovery phase [(33.25 ± 3.09), (16.13 ± 1.17) and (19.54 ± 1.43) points respectively] (P<0.05).

**Conclusions:**

DEACMP was accompanied by secondary brain injury, for which glial activation may be important. Serum S100B and GFAP levels may be related to prognosis.

## INTRODUCTION

Delayed encephalopathy after acute carbon monoxide poisoning (DEACMP) refers to a group of neuropsychiatric symptoms mainly including acute dementia that appears once again several days or even weeks of intermittent period (pseudo-recovery period) with completely or basically normal performance after rescue from ACMP.^1^,^2^ The most common pathological changes are diffuse demyelination of the white matter, mainly related to glial cell proliferation and tissue fibrosis. Brain tissues can be damaged through early and secondary injuries, inducing changes in the number and morphology of glial cells.^3^,^4^ The pathogenesis of DEACMP is similar. Therefore, whether secondary brain damage exists in DEACMP and whether the specific markers for glial injury can be used in early diagnosis and prognosis evaluation should be explored.

S100 protein is an acidic calcium-binding protein isolated from bovine brain for the first time by Moore in 1965. As a family member, S100B protein exists mainly in the cytosol of astrocytes in the central nervous system.^5^ When the central nervous system is damaged, S100B protein overflows from injured brain cells and enters the blood circulation through the blood-brain barrier, and its expression level is closely associated with the degree and prognosis of such damage.^6^-^8^ Glial fibrillary acidic protein (GFAP) is a specific acidic protein in the cytoplasm of astrocytes in the central nervous system. In recent years, it is well-documented that serum and cerebrospinal fluid GFAP levels are of great significance to the diagnosis, treatment and prognosis evaluation of neurological diseases.^9^-^11^

In this study, the serum levels of S100B and GFAP in patients with DEACMP in acute and recovery phases were measured and compared with those of patients without DE after ACMP, aiming to clarify their clinical significance for improving future diagnosis and treatment.

## METHODS

DEACMP patients who were hospitalized from November 2014 to February 2016 were selected.

### Inclusion criteria

Patients conforming to the DEACMP diagnostic criteria proposed by Zhao et al. were included.^12^ There were 66 eligible patients in total, including 36 males and 30 females aged between 43 and 80 years old, (58.17 ± 4.17) on average. We used the Glasgow Coma Scale for evaluation: Mild, 12-14 points; moderate, 9-12 points; severe, 3-8 points. All patients had disturbance of consciousness during ACMP (scores of Glasgow Coma scale: ≤8 points), which lasted for 2-72 h, (24.1 ± 8.7) h on average. Of all cases, 36 cases were ≤12 h, 28 were 13-48 h, and another two were >48 h. The intermittent period (referring to the time interval between the recovery of ACMP from coma and the occurrence of DE symptoms) lasted for 8-45 d, (20.1 ± 5.2) d on average. Among them, 38 cases were ≤20 d and 28 were ≥21 d. Brain CT examination disclosed that 8 cases were normal, 44 had low-density foci in the basal ganglia, and 50 had diffuse low-density shadows in the white matter. EEG examination showed 22 cases of severe diffuse abnormalities, 24 cases of moderate diffuse abnormalities, 6 cases of mild diffuse abnormalities, and 14 cases of focal abnormalities.

Meanwhile, 64 patients without DE after ACMP hospitalized in the same period were also selected. The enrolled patients conformed to the ACMP diagnostic criteria (China National Standard GB8781-88), all of whom had disturbance of consciousness during acute poisoning, with the serum carboxyhemoglobin levels ≥10%. All patients in the ACMP group received hyperbaric oxygen and routine therapies (vasodilators, neurotrophic drugs, etc.), and recovered after rescue. The follow-up time after recovery was ≥90 d, without the onset of DEACMP. The 64 patients included 34 males and 30 females who were aged between 42 and 80 years old, (57.11 ± 4.25) on average. The durations of coma owing to acute poisoning were 1-40 h, (8.7 ± 1.4) h on average. Of the 64 cases, 48 were ≤12 h and another 16 were 13-40 hour.

The baseline clinical data (e.g. age and gender) of the two groups were similar (P>0.05). This study has been approved by the ethics committee of our hospital (No. 20141120). All enrolled patients understood the purpose and contents of this study, signed the informed consent, and actively participated in the whole process.

### Clinical Evaluation

DEACMP patients were assessed by using activities of daily living (ADL) scale, information-memory-concentration test (IMCT) and Hasegawa’s dementia scale (HDS) on the day of blood sampling. All examinations were performed by the same attending physician in the Neurology Department.

### Sample Collection and Detection

Blood sampling was conducted for the DEACMP group in the acute phase the next day after hospitalization [disease courses: 5~14 d, (9.6 ± 2.0) d on average], and for the DEACMP group in the recovery phase before discharge from hospital [disease courses: 38~125 d, (63.4 ± 22.7) d on average]. The interval between two samplings was ≥30 d. For the ACMP group, blood sampling was carried out the next day after successful rescue and complete soberness. In detail, 5 ml of fasting non-anticoagulant blood was taken from the cubital vein of all patients at 6-8 am. After centrifugation, 2-3 ml of serum was collected and stored in a -80°C refrigerator for the detection of S100B and GFAP. Double antibody sandwich enzyme-linked immunosorbent assay was performed according to the kit (Shanghai Xinyu Biotechnology Co., Ltd., China) instructions.

### Statistical Analysis

All data were analyzed by SPSS 16.0. The quantitative data were expressed as (x ± SD). All data were subjected to the normality test, and those with variance homogeneity underwent within-group or paired test, or one-way analysis of variance. Inter-group comparisons were conducted by the LSD method. The data with variance heterogeneity were compared using the Dunnett’s T3 test. Correlation analysis was carried out with the Pearson linear correlation analysis for a bivariate process. P<0.05 was considered statistically significant.

## RESULTS

### Serum levels of S100B and GFAP of DEACMP group in the acute phase and ACMP group

Serum S100B and GFAP levels of the DEACMP group in the acute phase were significantly higher than those of the ACMP group (P<0.05) (Table-I).

**Table-I:** Serum levels of S100B and GFAP of DEACMP group in the acute phase and ACMP group (x ± SD, ng/mL)

Group	Case No.	S100B	GFAP
DEACMP group in the acute phase	66	0.59±0.11	227.67±12.43
ACMP group	64	0.48±0.10	178.91±11.47
T		5.960	23.225
P		0.000	0.000

### Serum levels of S100B and GFAP of DEACMP group in acute and recovery phases

Serum S100B and GFAP levels of the DEACMP group in the acute phase were significantly higher than those of the DEACMP group in the recovery phase (P<0.05) (Table-II). The serum S100B and GFAP levels of the DEACMP group were significantly positively correlated in both acute and recovery phases (r=0.432 in acute phase, P=0.007; r=0.378 in recovery phase, P=0.034).

**Table-II:** Serum levels of S100B and GFAP of DEACMP group in acute and recovery phases (x ± SD, ng/mL).

Group	Case No.	S100B	GFAP
DEACMP group in the acute phase	66	0.59±0.11	227.67±12.43
DEACMP group in the recovery phase	66	0.49±0.12	179.54±12.32
T		4.955	22.342
P		0.000	0.000

### Relationship between serum S100B, GFAP levels and prognosis in DEACMP group

After treatment, DEACMP patients were divided according to the treatment outcomes upon discharge. *Cured*: Disappearance of clinical symptoms, recovery of consciousness, and normal ability of daily living; significantly effective: significant alleviation of clinical symptoms, and basic recovery of intelligence, language and ability of daily living; effective: alleviation of clinical symptoms, but partly with movement disorder; ineffective: without any alleviation, with dementia, motor dysfunction and urinary and fecal incontinence, failure to handle daily living, as well as quitters. The serum S100B and GFAP levels of the ineffective DEACMP group in the acute phase were significantly higher than those of other groups (P<0.05) (Table-III). The results of cured, significantly effective and effective groups were similar (P>0.05).

**Table-III:** Serum S100B and GFAP levels of DEACMP groups in the acute phase with different treatment outcomes (x ± SD, ng/mL).

Group	Case No.	S100B	GFAP
Cured	20	0.50±0.09^abc^	167.67±11.83^abc^
Significantly effective	26	0.52±0.07^ab^	172.54±11.52^ab^
Effective	14	0.55±0.09^a^	179.98±11.78^a^
Ineffective	6	0.71±0.07	291.89±13.44
F		6.254	3.721
P		0.002	0.032

Compared with ineffective group,

a P<0.05; compared with effective group,

b P>0.05; compared with significantly effective group,

c P>0.05.

### ADL, HDS and IMCT scores of DEACMP group as well as correlations with S100B and GFAP levels

The ADL, HDS and IMCT scores of the DEACMP group in the acute phase were significantly different from those of the recovery phase (P<0.05) (Table-IV). Table-V shows that the scores are not correlated with S100B or GFAP level in both phases (P>0.05).

**Table-IV:** ACD, HDS and IMCT scores of DEACMP group in acute and recovery phases (x ± SD, point).

Group	ADL	HDS	IMCT
DEACMP group in the acute phase	45.12±3.12	7.98±1.02	9.61±1.41
DEACMP group in the recovery phase	33.25±3.09	16.13±1.17	19.54±1.43
T	21.960	42.656	40.170
P	0.000	0.000	0.000

**Table-V:** Correlations of ACD, HDS and IMCT scores with S100B and GFAP levels in acute and recovery phases.

Score	S100B		GFAP	

	r	P	r	P
ADL	0.035	0.454	0.170	0.214
HDS	-0.021	0.253	-0.191	0.201
IMCT	-0.091	0.168	-0.220	0.287

## DISCUSSION

CO is a protoplasmic toxin that can poison cells in systemic tissues. After inhalation, it forms stable carbonyl hemoglobin. The affinity of CO to hemoglobin is 240-fold that of O_2_, and the dissociation of carbonyl hemoglobin is only 1/3600 of oxyhemoglobin. As a non-cumulative toxin, CO can be gradually dissociated from carbonyl hemoglobin after contact and treatment, so it has no chronic toxic effects.^13^-^15^ Up to now, the pathogenesis for DEACMP remains unclear. Brvar et al. found in 38 CO poisoning cases that plasma S100B level was closely associated with CO poisoning.^16^ Animal experiments also proved that such level was an important parameter for evaluating the prognosis of ACMP. However, Rasmussen et al. reported that plasma S100B level barely increased after ACMP, without significant relationship. GFAP is an acidic protein (50-52 kDa) belonging to cytoskeletal protein, which is abundant and only expressed in astrocytes.^17^ Various brain damages including cerebral hemorrhage are all accompanied by elevation of serum GFAP level, as a marker for the damage of the central nervous system and its prognosis.^18^ GFAP and S100B can complementarily provide information for craniocerebral injury, severe brain damage and progressive secondary glia activities.

In this study, the DEACMP group in the acute phase had significantly higher S100B and GFAP levels than those of the ACMP group, suggesting that DEACMP indeed involved secondary injury and increase of the blood-brain barrier allowed the protein markers to enter the peripheral blood, leading to concentration elevation in the serum. Besides, serum S100B and GFAP levels in the recovery phase significantly decreased compared with those in the acute phase. Probably, active treatment well controlled the injury of cells in brain tissues. Given that the dynamic changes of serum S100B and GFAP levels were basically consistent with the variations of patient conditions and clinical treatment outcomes, these two biochemical indices can be employed to early diagnose brain damage and to determine the degree and prognosis. Meanwhile, serum S100B and GFAP levels were significantly positively correlated in both acute and recovery phases, indicating that individual use of an index was feasible.

Additionally, the significant increase of serum S100B and GFAP levels in DEACMP patients together with their significantly positive correlation suggested that delayed dysfunction or continuous death of glias after brain damage induced outflow of S100B and GFAP. The detailed mechanism for the role of glias in DEACMP-induced injury has seldom been studied hitherto. Possibly, glias dominantly participate in the onset of DEACMP after being activated.

In summary, DEACMP was accompanied by secondary brain injury, in which glial activation may play a key role. Serum S100B and GFAP levels may be related to prognosis. Nevertheless, we only detected the S100B and GFAP levels in the peripheral blood of DEACMP patients. In-depth studies are needed to clarify the pathophysiological mechanisms for DEACMP-induced secondary injury as well as the relationship between S100B and GFAP levels and the brain repair or plasticity. Furthermore, we will validate the specificity and sensitivity of S100B and GFAP for the diagnosis and prognosis evaluation of nervous system injuries.

### Authors’ Contributions

**CD & YZ**: Designed this study and prepared this manuscript.

**JM, ZS & WG:** Performed this study and analyzed clinical data.
